# CAUSAL ANALYSES OF PALLIATIVE CARE OUTCOMES USING OBSERVATIONAL DATA: A REVIEW OF CURRENT LITERATURE

**DOI:** 10.1093/geroni/igac059.756

**Published:** 2022-12-20

**Authors:** Narae Kim, Jingjing Jiang, Melissa Garrido, Mireille Jacobson, David Mockler, Peter May

**Affiliations:** University of Southern California, Los Angeles, California, United States; Trinity College Dublin, Dublin, Dublin, Ireland; Boston University School of Public Health, Boston, Massachusetts, United States; University of Southern California, Los Angeles, California, United States; University of Dublin, Dublin, Dublin, Ireland; Trinity College Dublin, Dublin, Dublin, Ireland

## Abstract

People with serious medical illnesses disproportionately account for health care spending, and the last year of life is typically the most costly. Economic evidence to inform improvement efforts in care for this population is long recognized as thin relative to policy importance. Palliative care studies rely heavily on routinely collected data since conducting randomized controlled trials with subjects nearing the end of life is particularly challenging; however, observational studies face high risk of bias. We conducted a systematic review of the peer-reviewed and grey literature to identify quasi-experimental studies evaluating palliative care’s effect on costs and health care utilization. Eligible study designs were those that controlled for unobserved confounding using causal inference methods (e.g. difference-in-differences). Eligible outcomes were costs, health care use, and quality-of-life. Among 806 search results, we included 17 studies: seven used difference-in-differences methods, five used interrupted time series analysis, and five used instrumental variables. Reporting quality was variable. Studies reported a general pattern of improved outcomes associated with palliative care. However, the incidence of studies finding a significant difference was lower than cohort studies that don’t attempt to control for unobserved confounding. Studies that don’t control for unobserved confounding may be overestimating true effects due to bias. Given the large volume of routine data collection in end-of-life care, there exists clear potential to increase the application of the causal inference methods to palliative care research. Such studies would strengthen the evidence base on an understudied topic and potentially inform other types of economic analysis in palliative care.

